# Legal Aspects of Pediatric Dentistry in India: A Narrative Review

**DOI:** 10.7759/cureus.102377

**Published:** 2026-01-27

**Authors:** Laresh N Mistry, Saba Kondkari, Sumeet Agarwal, Himmat Jaiswal, Sindhu P Pulaskar, Sayem A Mulla

**Affiliations:** 1 Pedodontics and Preventive Dentistry, Bharati Vidyapeeth (Deemed to be University) Dental College and Hospital, Navi Mumbai, IND; 2 Dentistry, Bharati Vidyapeeth (Deemed to be University) Dental College and Hospital, Navi Mumbai, IND; 3 Prosthodontics, Bharati Vidyapeeth (Deemed to be University) Dental College and Hospital, Navi Mumbai, IND; 4 Conservative Dentistry and Endodontics, Bharati Vidyapeeth (Deemed to be University) Dental College and Hospital, Navi Mumbai, IND; 5 Dentistry, Dentist and Independent Researcher, Kalyan, IND

**Keywords:** clinical pediatric dentistry, ethical practice, legal issues, medical-legal, medico-legal issues

## Abstract

The interface between pediatric dentistry and law in India is increasingly significant as dental practitioners face expanding medico-legal accountability, consumer rights awareness, and evolving digital challenges. This paper critically explores the regulatory frameworks, ethical obligations, and medico-legal considerations that shape pediatric dental practice in India. It highlights statutory obligations under the Dentists Act, 1948, the Clinical Establishments Act, 2010, the Consumer Protection Act, 2019, and the Protection of Children from Sexual Offences (POCSO) Act, 2012, while addressing consent, confidentiality, digital documentation, and forensic responsibilities. The discussion underscores the necessity of preventive legal literacy, meticulous record-keeping, and ethical vigilance to foster legally resilient and patient-centered pediatric oral care.

## Introduction and background

The legal and ethical dimensions of pediatric dentistry have gained substantial prominence in recent years, particularly as the Indian dental profession transitions toward greater accountability and patient rights awareness [1]. Pediatric dentists occupy a unique position, entrusted not only with managing the oral health of minors but also with navigating sensitive domains of consent, confidentiality, and mandatory reporting [2]. The Dentists Act, 1948, remains the cornerstone of professional regulation, establishing the Dental Council of India (DCI) as the statutory body overseeing education and professional standards [3].

The inclusion of dentistry under the Consumer Protection Act, 2019, redefined patient-clinician relationships, subjecting practitioners to civil liability for negligence or deficiency in service [4]. Concurrently, the Clinical Establishments (Registration and Regulation) Act, 2010, mandates adherence to standards of hygiene, infrastructure, and documentation, directly impacting pediatric dental clinics [5].

Digitalization and the National Health Authority’s Health Data Management Policy (2020) have added layers of responsibility regarding data privacy and informed consent. At the same time, pediatric dentists remain critical partners in child protection under the Protection of Children from Sexual Offences (POCSO) Act, 2012, and Juvenile Justice Act, 2015, both of which emphasize mandatory reporting and forensic collaboration [6].

Thus, pediatric dentistry in India operates within a complex legal ecosystem integrating statutory mandates, ethical principles, and emerging technological imperatives. Understanding these dimensions is vital for fostering both professional resilience and child-centered care.

## Review

Methodology

In order to find pertinent research analyzing the legal, ethical, and medico-legal aspects of pediatric dentistry with a focus on the Indian regulatory context, a thorough literature search was carried out. From the database's creation until December 31, 2025, electronic databases such as PubMed/MEDLINE, Scopus, Web of Science, and Google Scholar were searched; the last search was carried out in January 2026. Only publications written in English were taken into account. Medical Subject Headings (MeSH) and free-text terms, such as "Pediatric Dentistry," "Paediatric Dentistry," "Dental Ethics," "Medical Law," "Legislation," "Informed Consent," "Negligence," "Consumer Protection," "Child Abuse," and "India," were combined using Boolean operators as part of the search strategy. To find further sources, the reference lists of pertinent policy papers and suitable publications were manually checked.

Studies that addressed legal, ethical, or medico-legal concerns pertinent to pediatric dentistry practice, such as consent, confidentiality, professional responsibility, child protection, documentation, and regulatory compliance, were included, especially if they dealt with Indian or similar legal frameworks. Non-English publications, abstracts without complete text, clinical technique-only articles without legal or ethical debate, and non-scholarly opinion pieces were all disqualified. After screening titles and abstracts, two independent reviewers evaluated possibly suitable papers in full. Disagreements were settled by discussion and consensus. The final synthesis had a total of 24 articles that satisfied the inclusion criteria. The results were compiled conceptually rather than statistically due to the diversity and normative character of the legal and policy literature.

Regulatory and legal framework governing pediatric dental practice in India

Pediatric dental practice in India is governed by a multilayered legal and regulatory structure designed to safeguard both practitioners and patients [7]. The Dentists Act of 1948 empowers the DCI to regulate dental education, licensure, and ethical conduct. The DCI’s 2023 revised curriculum emphasizes preventive and patient-safety competencies within pediatric dentistry [8]. The Clinical Establishments (Registration and Regulation) Act of 2010 requires every dental facility to meet specified standards concerning infrastructure, infection control, and record maintenance. Violations may constitute negligence under the Consumer Protection Act of 2019, which identifies dental patients as consumers entitled to redress for deficient services [9].

In pediatric dentistry, consent is important because minors lack legal capacity. Guardians must provide written consent for invasive procedures, and failure to obtain or record it may amount to professional misconduct [10]. Judicial precedents reinforce that practitioners are liable when their conduct deviates from that of an ordinarily competent peer. The Health Data Management Policy (2020) expands the regulatory domain to electronic records and tele-dentistry, introducing enforceable standards of privacy and consent. International frameworks, such as those by the General Dental Council (UK) and American Academy of Pediatric Dentistry (AAPD), similarly emphasize patient safety, consent, and continuing education benchmarks that can enrich Indian dental jurisprudence [11]. Collectively, these statutory and ethical instruments create a unified structure that anchors pediatric dental care within the principles of legality, accountability, and transparency.

Consent, confidentiality, and liability in pediatric dentistry

Consent and confidentiality form the ethical and legal core of pediatric dental care. Minors lack full contractual capacity under the Indian Contract Act of 1872; hence, legal guardians must provide consent for treatment. Consent must be informed, detailing the nature, risks, alternatives, and benefits. For procedures like pulpectomy or sedation, written consent is mandatory. Ethically, children should also provide assent, a willingness (show or consent) to undergo treatment, even if not legally binding. This aligns with AAPD guidelines emphasizing respect for a child’s developing autonomy [12].

Confidentiality obligations extend to all patient data, strengthened by the Health Data Management Policy (2020) requiring explicit consent for data handling and sharing. However, confidentiality yields to legal duties under the POCSO Act (2012), which mandates reporting suspected sexual abuse. Non-reporting attracts criminal penalties [13].

Liability in pediatric dentistry spans both civil and criminal dimensions. Negligence is established when care falls below accepted professional standards. Comprehensive documentation covering consent, treatment details, and follow-up is thus indispensable. The Consumer Protection Act, 2019, further empowers patients to claim redress for deficiencies, necessitating meticulous record-keeping and communication [14]. Pediatric dentists must therefore maintain a balance between parental communication, child rights, and procedural diligence to ensure both ethical integrity and legal defensibility.

Child protection, abuse and neglect, forensic, and ethical responsibilities

Pediatric dentists are often the first professionals to detect signs of child abuse or neglect. Oral injuries manifest in 65-75% of physically abused children [15], placing the onus on dentists to identify and report suspected cases under the POCSO Act, 2012, and the Juvenile Justice Act, 2015. Failure to report may invite criminal prosecution under Section 21 of the POCSO Act. Recognition of intraoral injuries such as bruises, burns, torn labial frena, or unexplained fractures requires clinical vigilance. Proper documentation, including photographs and radiographs, serves both clinical and forensic purposes. The DCI’s curriculum now mandates exposure to forensic odontology to enhance competence in legal documentation. Maintaining the chain of custody for evidence ensures admissibility in legal proceedings [16].

Ethically, pediatric dentists must balance confidentiality with their duty to protect vulnerable children. While data privacy laws protect patient information, ethical principles such as beneficence and non-maleficence justify disclosure to authorities in suspected abuse cases [17]. The AAPD’s Policy on Ethical Responsibility and Patient Safety (2022) reinforces minimal restraint, informed parental consent, and prioritizing child welfare [18]. With emerging technologies, such as digital imaging and artificial intelligence (AI)-assisted diagnostics, dentists must also navigate issues of consent for photography, image storage, and data sharing. Ethical vigilance thus extends into digital domains, demanding a convergence of forensic accuracy, legal compliance, and moral responsibility [19].

Documentation, digital dentistry, and emerging legal frontiers

Meticulous documentation remains the strongest defense against malpractice claims. Accurate charting of diagnosis, treatment plans, and informed consent protects both patient and practitioner [20]. The Clinical Establishments Act, 2010, mandates standardized record maintenance, including radiographs and follow-up notes. Digital transformation has introduced complex responsibilities related to data protection. The Health Data Management Policy (2020) enforces explicit consent for digital record use and mandates data encryption, storage security, and access control [21].

AI tools used in caries detection and growth analysis introduce new medico-legal dimensions. Despite AI’s diagnostic assistance, final accountability remains with the clinician. The proposed Digital Information Security in Healthcare Act (DISHA) aims to strengthen privacy and accountability for such technologies [22]. Future pediatric dental practice must integrate innovation with compliance, ensuring that digital adoption aligns with ethical documentation, patient consent, and statutory regulation.

Medico-legal preparedness and future directions in pediatric dentistry

Preparedness in medico-legal matters is now central to professional sustainability in pediatric dentistry. Increasing consumer awareness has intensified legal scrutiny, necessitating preventive strategies such as transparent communication, consent renewal, and systematic documentation [23]. Regular audits, standardized consent templates, and medico-legal training foster readiness and professional accountability. The DCI (2023) now advocates legal and ethical literacy within postgraduate programs [9]. Continuing professional development (CPD) in documentation, ethics, and risk management enhances resilience against potential litigation.

Looking forward, digital integration, AI-based diagnostics, and international harmonization with AAPD and FDI World Dental Federation frameworks will shape the future of pediatric dental jurisprudence. The proposed DISHA legislation will expand legal accountability to digital practice, requiring practitioners to adopt secure systems and transparent consent mechanisms [22]. Ultimately, legal awareness, ethical empathy, and self-regulation are the foundations of a legally resilient pediatric dental profession in India, one that prioritizes both patient safety and professional integrity [24].

## Conclusions

The practice of pediatric dentistry in India is intricately bound to evolving legal and ethical expectations. Statutory instruments such as the Dentists Act, 1948, Clinical Establishments Act, 2010, Consumer Protection Act, 2019, and POCSO Act, 2012, collectively define the contours of professional responsibility. Ethical imperatives surrounding consent, confidentiality, and child protection complement these legal frameworks, guiding clinicians toward holistic and defensible care. As digital technologies transform practice patterns, new accountability challenges emerge, underscoring the need for continuous education in medico-legal and ethical domains. Pediatric dentists, as custodians of children’s welfare, must integrate compassion with compliance to ensure a safe, transparent, and future-ready practice environment.
